# Potential use of bacterial community succession for estimating post-mortem interval as revealed by high-throughput sequencing

**DOI:** 10.1038/srep24197

**Published:** 2016-04-07

**Authors:** Juanjuan Guo, Xiaoliang Fu, Huidan Liao, Zhenyu Hu, Lingling Long, Weitao Yan, Yanjun Ding, Lagabaiyila Zha, Yadong Guo, Jie Yan, Yunfeng Chang, Jifeng Cai

**Affiliations:** 1 Department of Forensic Science, School of Basic Medical Sciences, Central South University, Changsha, Hunan 410013, China; 2 Xiangya School of Medicine, Central South University, Changsha, Hunan 410013, China

## Abstract

Decomposition is a complex process involving the interaction of both biotic and abiotic factors. Microbes play a critical role in the process of carrion decomposition. In this study, we analysed bacterial communities from live rats and rat remains decomposed under natural conditions, or excluding sarcosaphagous insect interference, in China using Illumina MiSeq sequencing of 16S rRNA gene amplicons. A total of 1,394,842 high-quality sequences and 1,938 singleton operational taxonomic units were obtained. Bacterial communities showed notable variation in relative abundance and became more similar to each other across body sites during the decomposition process. As decomposition progressed, Proteobacteria (mostly Gammaproteobacteria) became the predominant phylum in both the buccal cavity and rectum, while Firmicutes and Bacteroidetes in the mouth and rectum, respectively, gradually decreased. In particular, the arrival and oviposition of sarcosaphagous insects had no obvious influence on bacterial taxa composition, but accelerated the loss of biomass. In contrast to the rectum, the microbial community structure in the buccal cavity of live rats differed considerably from that of rats immediately after death. Although this research indicates that bacterial communities can be used as a “microbial clock” for the estimation of post-mortem interval, further work is required to better understand this concept.

Decomposition is a complex process involving both biotic (cellular enzymes of the cadaver, bacteria, fungi, protozoa, insects and carnivores) and abiotic (weather, climate and humidity) factors. The decomposition of vertebrates can be categorised into stages based on the physical appearance of the remains and the associated arthropod arrival patterns. A body commonly passes through several stages as decomposition progresses. Vass^1^ described the progression of human decomposition as taking place in four stages: fresh (autolysis), bloat (putrefaction), decay (putrefaction and carnivores) and dry (diagenesis). Wolff *et al*.^2^ observed five different stages of decomposition: fresh, bloated, active decay, advanced decay and dry, which were determined by the physical and body temperature changes of the carcass. Decomposition begins approximately 4 min after death with a process called autolysis or self-digestion^3^. Enzymes from within the dead cells of the cadaver begin to release and damage cellular components and metabolites^4^,^5^. Shortly after death, intrinsic bacteria also begin to digest the body from the inside. When vertebrate scavengers are prohibited, the loss of biomass is driven primarily by immature Diptera and microbes^6^. If sarcosaphagous insect colonisers have access to a corpse, they use the carrion as a nutrient source and oviposition site, with blowfly larvae alone capable of consuming over 80% of the available material^7^.

Post-mortem interval (PMI) estimation is one of the most important and difficult tasks in forensic practice. Traditionally, estimation of PMI has relied on the physical changes that occur after death, including algor mortis, livor mortis, rigor mortis and supravital activity, which are well recognised as early post-mortem phenomena^8^,^9^. However, because these states do not continue into the late post-mortem period, they can only provide a rough estimation of the PMI. In recent years, additional techniques have been employed for estimating the PMI of animal remains, including examining thanatochemistry^10^,^11^,^12^, DNA/RNA degradation^13^,^14^ and forensic entomology^15^,^16^. However, none of these methods are widely accepted by forensic science experts, and accurate PMI estimation remains a challenge.

From the viewpoint of forensic entomologists, the PMI can be divided into two principal parts: the pre-colonisation interval (pre-CI), which spans the period between the time of death and the arrival of sarcosaphagous insects, and the post-colonisation interval (post-CI), which is applied to the broad time range between initial insect colonisation and body corruption^17^. Forensic entomologists can estimate the post-CI based on the presence of specific families and species of insects that feed on the corpse (mainly those in the orders Diptera and Coleoptera)^18^. In contrast, in the pre-CI or in colder months when insects are not active, the metabolic activities of microbes constitute the major components of the decomposition process.

Past studies of microbes were primarily based on culture-dependent techniques^19^,^20^,^21^. Using a mouse model of decomposition, Melvin *et al*.^19^ demonstrated that *Staphylococcus* species were the first microorganisms to migrate from the small intestine, followed by coliforms and *Candida*, and then anaerobic bacteria. However, it is estimated that up to 99% of bacterial species found in nature cannot be cultured by conventional means^22^, and approximately 80% of bacteria identified by metagenomic sequencing were considered noncultivable^23^. The rapid development of parallel high-throughput sequencing technologies has resulted in the commercialisation and widespread adoption of next-generation sequencing technologies for analysis of bacterial community structure. As such, there are numerous studies incorporating bacterial community analysis in the evaluation of carrion decomposition processes and estimating PMI in terrestrial ecosystems^6^,^24^,^25^,^26^.

Pechal *et al*.^26^ defined the epinecrotic community as those organisms residing, or moving, on the surface of decomposing remains, including the skin and mucous membranes of cavities (e.g., mouth). This community is primarily made up of prokaryotes, protists and fungi. In this study, we used high-throughput sequencing to investigate epinecrotic community composition and succession during the decomposition process for the purpose of estimating PMI. These data may be able to uncover the underlying microbial ecology of corpse decomposition, as well as provide additional information to aid in finding a linear relationship for using microbes as a potential tool for forensic purposes. We conducted exploratory investigations of the epinecrotic community from live rats and their decomposed cadavers under both sarcosaphagous insect exclusion and access conditions. To the best of our knowledge, this is the first report of the bacterial biodiversity in decomposing rats under terrestrial conditions in China.

## Results

### Progression of decomposition

The average daily air temperature was 25.74 ± 1.80 °C (s.d.), and ranged from 22.71 to 27.67 °C. The mean daily air humidity was 73.28 ± 10.65% (s.d.), and ranged from 60.29 to 89.38% during the study. And accumulated degree hours (ADHs) increased linearly throughout decomposition (Supplementary Fig. S1). Rats in the sarcosaphagous insect exclusion group (group A) merely progressed to the active decay stage of decomposition during the 8-day observation period, while rats in the sarcosaphagous insect access group (group B) progressed through all stages of decomposition. The fresh stage began at the time of death, with a sharp decrease in body temperature, corneal opacity, cyanosis and rigor mortis. The bloat stage occurred 12 h post-mortem in group B (333 ADH) and 18 h post-mortem (480 ADH) in group A, and was characterised by the expansion of the torso because of gas accumulation. The active decay stage occurred 2 days post-mortem (1268 ADH) in group B and 4 days post-mortem (2445 ADH) in group A, and was indicated by strong odour, abdominal rupture and putrefactive fluid leakage. The advanced decay stage was initiated 4 days post-mortem (2445 ADH) in group B, and was characterised by an absence of odour, the removal of the majority of the soft tissue and the presence of third instar Calliphoridae and Sarcophagidae larvae. The dry stage occurred 6 days post-mortem (3624 ADH) in group B and represented the end of decomposition, with the vast majority of soft tissue removed and only remains of skin and bones still present.

In group B, an adult *Lucilia cuprina* (Diptera: Calliphoridae) was the first insect to arrive on the carcass, 8 h post-mortem (230 ADH). Adults of the families Calliphoridae and Sarcophagidae oviposited in the natural orifices (e.g. oral cavity, nares, external meatus and anus), on the neck and on the side of the carcass touching the ground during the bloat and active decay stage. Second and third instar Calliphoridae and Sarcophagidae larvae were found during both the active and advanced decay, mainly located in large masses that were concentrated towards the side of the carcass that was touching the ground. The great abundance (ranging from 66.19% to 85.71%) of adult Calliphoridae were noted in the dipterans collected from the fresh stage to advanced decay stage. The larvae dispersed from the carcass in the dry stage to pupate, leaving only a few adult Muscidae and Silphidae (Supplementary Tables S1 and S2).

### Overview of sequencing analysis

A total of 1,413,910 raw sequences were generated in the current study. After sequence trimming, quality filtering and removal of chimeras, 1,394,842 high-quality sequences remained, with an average length of 253 bases. The mean number of sequences per sample was 41,676 ± 16,976 (s.d.). The rarefaction curves indicated that species representation in each sample had approached the plateau phase, and it was unlikely that more bacteria would be detected with additional sequencing efforts (Supplementary Fig. S2). These high-quality sequences were clustered into 10,977 operational taxonomic units (OTUs) by the UPARSE pipeline^27^ using a threshold of 97% identity, with an average of 333 OTUs per sample. In total, 1,938 OTUs were singletons. We classified 99.92 ± 0.11% (s.d.) of high-quality sequences to the phylum level, 98.00 ± 2.44% (s.d.) to the family level, 84.51 ± 13.44% (s.d.) to the genus level and 32.71 ± 27.74% (s.d.) to the species level. The results showed bacteria belonging to 48 phyla, 119 classes, 175 orders, 228 families, 278 genera and 75 species. Detailed characteristics of each sample are listed in Table 1, and all raw sequences were deposited in the Sequence Read Archive (accession number SRP063690).

### Bacterial diversity before and after death

**Figure 1:**
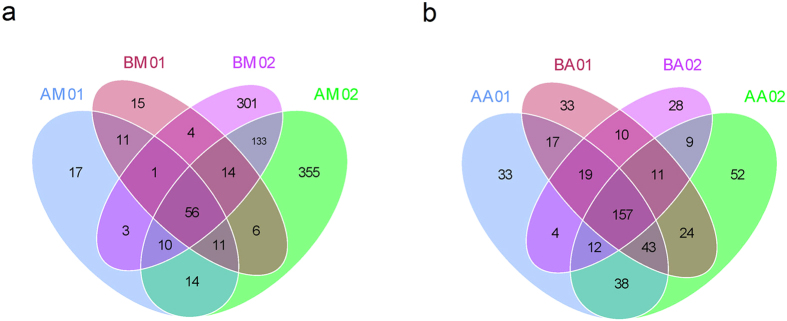
Shared operational taxonomic unit (OTU) analysis of the different communities. Venn diagrams showing the unique and shared OTUs in the different communities, (**a**) for the AM01, AM02, BM01 and BM02 communities, and (**b**) for the AA01, AA02, BA01 and BA02 communities. Sample names refer to samples as described in Table 1.

The OTU number and alpha diversity metrics for buccal cavity samples rapidly increased immediately after death compared with the samples collected from live rats (Table 1). A Venn diagram was used to compare the similarities and differences between the communities in the different samples (Fig. 1). The AM01, AM02, BM01 and BM02 communities had 56 OTUs in common (Fig. 1a), with the common OTUs comprising 98.53%, 69.49%, 98.46% and 77.89% of the sequences in the AM01, AM02, BM01 and BM02 communities, respectively (Table 2). The AA01, AA02, BA01 and BA02 communities had 157 OTUs in common (Fig. 1b), with the common OTUs comprising 94.86%, 94.90%, 96.69% and 98.25% of the sequences in the AA01, AA02, BA01 and BA02 communities, respectively (Table 2).

### Bacterial community composition and structure succession analysis

**Figure 2:**
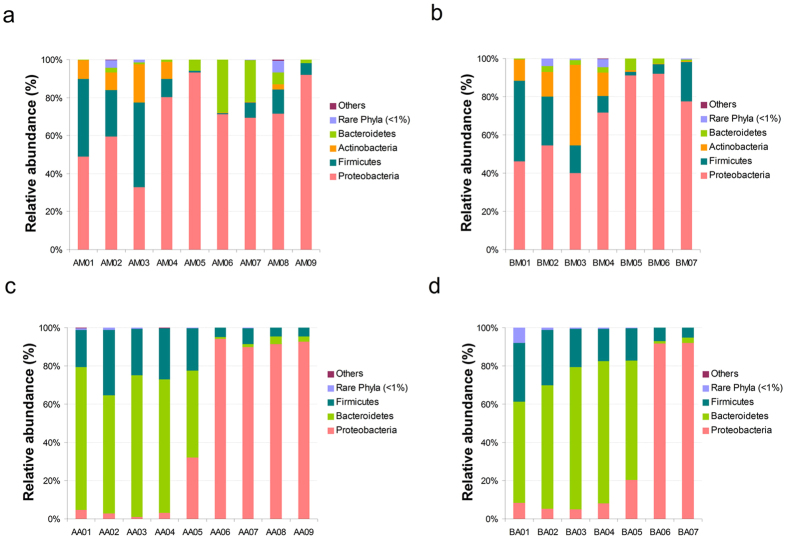
Bacterial community structure variation during decomposition at the phylum level. Relative abundance of bacterial phyla during decomposition in the buccal cavity in group A (**a**), buccal cavity in group B (**b**), rectum in group A (**c**) and rectum in group B (**d**). Sample names refer to samples as described in Table 1.

**Figure 3:**
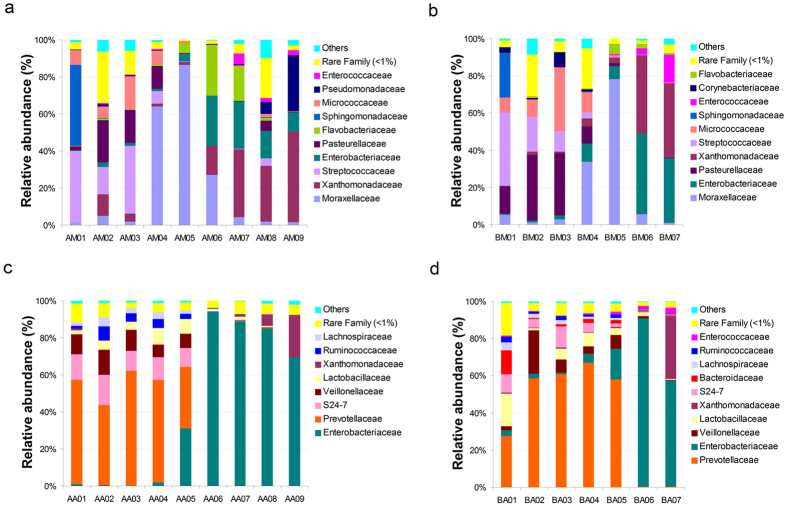
Bacterial community structure variation during decomposition at the family level. Relative abundance of bacterial families during decomposition in the buccal cavity in group A (**a**), buccal cavity in group B (**b**), rectum in group A (**c**) and rectum in group B (**d**). Sample names refer to samples as described in Table 1.

To identify bacterial community structure succession during decomposition, the 16S rRNA sequences were classified at both the phylum and the family levels. There were notable trends and changes in the relative abundance of the different bacterial taxa in the buccal cavities and rectums of the rat corpses throughout the decomposition process (Figs 2 and 3). In the buccal cavity, Proteobacteria was the dominant phylum (71.18 ± 19.31% (s.d.) in group A and 71.83 ± 20.60% (s.d.) in group B) as decomposition progressed, except in the 4-h post-mortem sample, where Firmicutes was the dominant taxon (44.40%) in group A and Actinobacteria became dominant (42.1%) in group B. Moraxellaceae increased in abundance in both groups to become the most abundant taxon on day 1 post-mortem, but gradually decreased thereafter. The abundance of Xanthomonadaceae gradually increased as decomposition progressed, and it became the dominant taxon from day 3 post-mortem onwards. The abundance of Enterobacteriaceae dramatically increased on day 2 post-mortem, but then decreased from day 4 onwards, while the abundance of Streptococcaceae and Pasteurellaceae gradually decreased throughout decomposition. Interestingly, the abundance of Pseudomonadaceae rapidly increased on day 6 post-mortem in group A. In the rectum samples of both groups, Bacteroidetes and Firmicutes were the predominant phyla until 2 days post-mortem, at which point Proteobacteria became the most abundant phylum. Similarly, Enterobacteriaceae took over from Prevotellaceae as the predominant family 2 days post-mortem.

There was a significant non-linear relationship between bacterial taxon richness and ADH. The non-linear model was a better fit based on the data points. Cubic is one of the non-linear forms, and it had the highest coefficient of determination (ranging from 0.308 to 0.351) compared to the other non-linear equations in non-linear form. OTU numbers, Chao1, Observed Species and Shannon index values declined prior to approximately 1600 ADH (2.5 days post-mortem), increased in the period from 1600 to 3624 ADH (6 days post-mortem) and finally decreased thereafter (Supplementary Fig. S3).

### Clustering patterns of samples from the buccal cavity and rectum during decomposition

**Figure 4:**
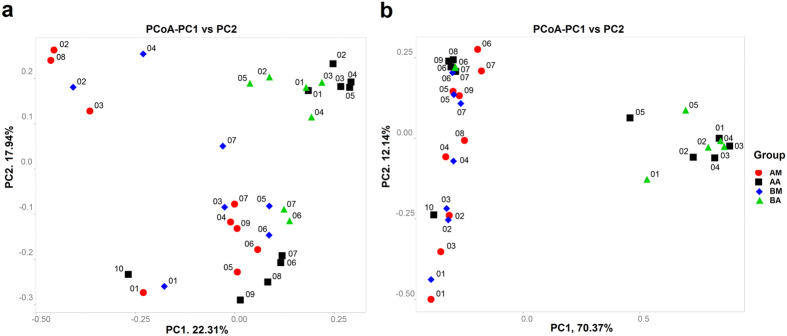
Two-dimensional principal coordinates analysis (PCoA) plot of unweighted (**a**) and weighted (**b**) unifrac distance matrices for buccal cavity and rectum samples during decomposition. The bacterial community of the buccal cavity from rat carcasses in group A (red circle) and in group B (blue diamond) and the bacterial community of the rectum from rat carcasses in group A (black square) and in group B (green triangle) were represented. Sample names refer to samples as described in Table 1.

**Figure 5:**
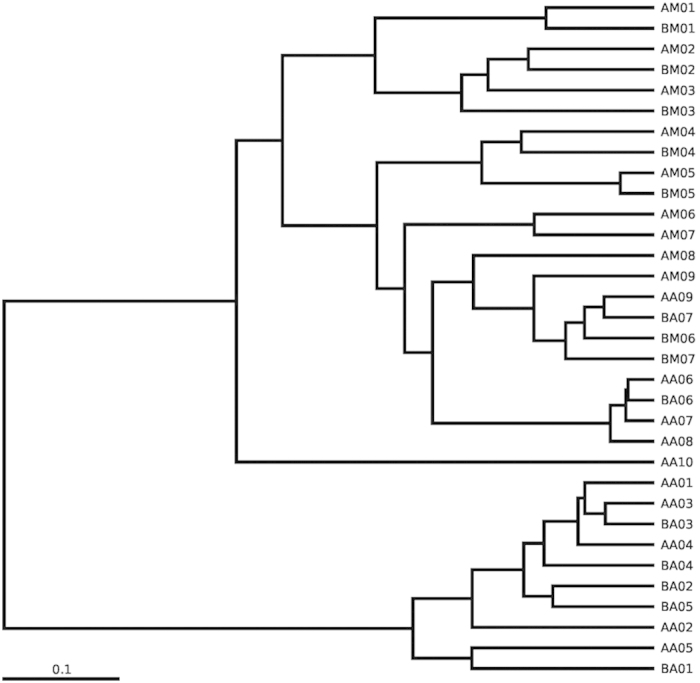
Hierarchical clustering of buccal cavity and rectum samples by unweighted pair group method with arithmetic mean (UPGMA) according to their weighted unifrac matrix. Sample names refer to samples as described in Table 1.

Principal coordinate analysis (PCoA) revealed the decomposition pattern in two-dimensional space for the unweighted and weighted unifrac distances (Fig. 4). According to the unweighted unifrac PCoA, samples collected from the buccal cavity of live rats formed a unique cluster, separate from the other samples (Fig. 4a). According to principal coordinate 1 (PC1) and PC2 analysis (22.31% and 17.94% of variance explained, respectively), the microbial communities of the buccal cavity and rectum were separated in the early PMI and clustered in the late PMI. The separation between samples across body sites in the early PMI and similarity in the late PMI were more notable on the weighted unifrac-based plot. According to weighted unifrac PCoA, the buccal cavity samples in early PMI, the rectum samples in early PMI and the microbial communities (both buccal cavity and rectum samples) in late PMI were grouped into three distinct clusters based on PC1 and PC2 analysis (70.37% and 5.23% of variance explained, respectively; Fig. 4b). Sample AA10, a rectum sample from rats in group A, clustered separately from other rectum samples and showed a different pattern of relative abundance (This might have been caused by sampling error or poor sample quality). The hierarchical clustering by unweighted pair group method with arithmetic mean (UPGMA) was used to analyse samples according to their weighted unifrac matrix (Fig. 5). This analysis revealed similar results to the weighted unifrac PCoA. The buccal cavity and rectum samples collected in the early decomposition stage formed distinct clusters, while the samples from the two body sites collected in the late decomposition stage were more similar.

### Differences between samples in the insect exclusion and access groups

Differences in the community composition between groups A and B were tested using the Analysis of Similarities description (Supplementary Tables S3 and S4). Although several taxa showed significant differences between the two groups, they were present in low abundance (mostly < 0.1%). For the buccal cavity samples, the genera *Escherichia, Wautersiella* and *Collinsella* were more prevalent in group B, and *Photobacterium, Facklamia, Nitrospina* and *Anaerotruncus* only appeared in group B. *Deinococcus, Methylocaldum, Spirochaeta, Halanaerobium* and *Acholeplasma* were absent from group B. For the rectum samples, the genera *Stenotrophomonas, Sutterella* and *Bifidobacterium* were enriched in group B, while *Thiobacillus, Wautersiella, Sulfurimonas* and *Lachnospira* were absent from group A. The abundance of *Anaerovibrio* and *Collinsella* was lower in group B than in group A. *Clostridium, Adlercreutzia* and *Caulobacter* were absent from group B.

## Discussion

In our study, the dominant phyla in the buccal cavity of the live rats were Proteobacteria, Firmicutes and Actinobacteria (Fig. 2), and the dominant genera were *Sphingomonas* and *Streptococcus* (Supplementary Fig. S4). The dominant rectal phyla of the live rats were Bacteroidetes and Firmicutes (Fig. 2), and the dominant genera were *Prevotella* and *Bacteroides* (Supplementary Fig. S4). These dominant phyla and genera were similar to those in the mouth and stool of healthy humans, as reported by the Human Microbiome Project (HMP)^28^. The HMP observed that human stool harbours a rich microbiome, while the oral cavity is more limited^28^. We observed the same phenomenon in samples collected from live rats (Table 1). In addition to having similar decay processes and mechanisms, the microbial communities of Sprague Dawley rats were similar to those of humans, indicating that such animals are appropriate models for human decomposition studies. Using a rat model, we were able to perform replicate experiments with a large number of samples to minimize experimental error and assess to what extent intra-individual variation in microbiota occurred during decomposition^25^.

More bacterial taxa were found in the buccal cavity of rats immediately after death than were present in live rats; however, there was no obvious variation pre- and immediately post-mortem in the rectum samples. Compared with the anus, the mouth is more exposed to microorganisms from external body locations (e.g. skin and hair) or the environment (e.g. soil and air). Saliva, which shows antimicrobial activity, ceases to be produced after death, contributing to increased bacterial survival. Interestingly, Sphingomonadaceae, one of the dominant families in the buccal cavity of live rats, showed a sharp decline in abundance immediately after death. Aerobic Gram-negative bacterial taxa of the family Sphingomonadaceae were usually reported to be isolated from different environments (e.g. soils, lakes and oceans). Lee *et al*.^29^ isolated several bacterial strains belonging to the genus *Sphingomonas* from the live mouse oral cavity as well.

Our data demonstrated that the relative abundance of dominant phyla Proteobacteria, Firmicutes, Bacteroidetes and Actinobacteria showed notable variation during the process of decomposition, which may aid in PMI estimation. In contrast to a previous report^26^, Proteobacteria and Moraxellaceae, the predominant phylum and family in buccal cavity samples, increased in abundance on day 1 post-mortem but slightly declined thereafter, while Firmicutes gradually decreased. The remaining dominant families (Xanthomonadaceae, Streptococcaceae, Enterobacteriaceae and Pasteurellaceae) showed changes in abundance that were consistent with the previous report^26^. In addition, Actinobacteria were prevalent members of the buccal cavity bacterial communities 4 h post-mortem, becoming even more abundant than Bacteriodetes in group B samples. Bacteroidetes and Firmicutes, the dominant phyla in rectum samples on day 1 post-mortem, were replaced by Proteobacteria from day 2 post-mortem onwards. Similarly, the most prevalent family on day 1 post-mortem was Prevotellaceae, which was replaced by Enterobacteriaceae on day 2 post-mortem. Metcalf *et al*.^25^ also found that Enterobacteriaceae, widely recognized as opportunistic pathogens associated with sewage and animal matter, became more abundant after rupture in the decay stage^25^. Proteobacteria are commonly associated with the spoiling of meat, and have been found on the hides of slaughtered animals^30^. Bacteroidetes and Firmicutes have been reported as the two main phyla in the human gut (intestine, rectum and cecum) and in faecal samples^23^,^28^,^31^,^32^,^33^. Actinobacteria are widely distributed in both terrestrial and aquatic ecosystems, especially in soil, where they play a crucial role in the recycling of refractory biomaterials through decomposition and humus formation^34^. We also found that *Proteus* and *Ignatzschineria* of the Gammaproteobacteria class were predominant both in the buccal cavity and the rectum in the late stages of decomposition (Supplementary Fig. S4). The Gammaproteobacteria display diverse metabolic capabilities, and are involved in the breakdown of more complex molecules^21^. Our results supported the theory that Gammaproteobacteria may be an important contributor to the process of decomposition.

Previous studies have shown that oral communities differ from gastrointestinal tract communities in healthy people and human cadavers in the bloat stage of decomposition^24^,^28^. However, in this study, we observed a separation of samples from the buccal cavity and rectum in the early PMI, whereas samples from the two body sites clustered together in the late PMI. Pechal *et al*.^26^ monitored swine decomposition under natural conditions and confirmed a significant negative linear relationship over time for overall phylum and family taxon richness. We also found a significant fluctuating non-linear relationship (with a general trend of descent) for overall bacterial taxon richness over the course of decomposition. Tuomisto *et al*.^35^ observed that the amount of bacterial DNA remained quite stable with time elapsed post-mortem, except for a substantial increase on day 5 post-mortem, during autopsies of rectum from male human. Those results indicated that the number of bacteria increased while the richness declined during the decomposition process. A shift from aerobic (*Streptococcus*) to anaerobic (*Proteus* and *Ignatzschineria*) bacteria was also observed in the bacterial communities, which was similar to previous studies^24^,^26^.

We noted an appreciable number of unclassified reads at the genus level in both the buccal cavity and rectum samples during the early PMI, and in the buccal cavity samples from rats decomposed in the absence of insects since the active decay stage. Previous study^26^ has produced very few unclassified reads from samples collected 1–3 days after death (Supplementary Fig. S5). Previous studies also concluded that approximately 80% of bacteria identified by metagenomic sequencing were noncultivable^23^. Although a limited number of unclassified sequences can result from PCR errors or sequencing artefacts, such an abundance of unclassified reads suggests a significant presence of novel bacterial taxa^36^. Potential novel species are assumed when there is a significant difference between the phenotypic characteristics and/or 16S rRNA sequences (more than 0.5% difference) of the unknown bacterium and those of the most closely related ones^37^,^38^. Many factors can influence the bacterial communities detected in and on a cadaver, including the individual’s starting microbiome, the decomposition environment of the cadaver, sample collection methodology and even differences in swab pressure during sample collection. Compared with previous studies, we sampled more time points on the first day post-mortem, and identified fluctuations in microbial abundance over time (especially in the Actinobacteria and Micrococcaceae). To establish the bacterial succession patterns during decomposition, and assess the potential of this information for application as a forensic tool, further study is required to test more time points and compile a larger database of necrobiome communities.

In the current study, the cadavers in the sarcosaphagous insect exclusion group (group A) took longer to decompose than those in the insect access group (group B). However, the arrival and oviposition of sarcosaphagous insects had no obvious influence on bacterial taxa during decomposition. Although there were some significant differences in the abundance of specific taxa between the sarcosaphagous insect access and exclusion groups, the abundance of these taxa was low and could generally be ignored. Symbiotic insects may benefit from the microbes already resident in the carcass, while competitors would be introduced from newly arrived insects and microbes. Other microorganisms transported to a carcass or corpse via flies could affect taxon richness through decomposition processes such as competition^26^. Blowflies may directly affect microbial species by initiating immune responses after pathogen detection^39^,^40^, and producing secretions with antibiotic properties^41^,^42^. Furthermore, fly larvae can obtain amino acids from a variety of bacteria. A previous study illustrated that bacteria or their metabolic products are essential nutrients for house fly maggot growth, and a wide variety of bacteria can contribute to the suitability of an organic substrate for maggot growth^43^. Additionally, carrion microbial communities influence the arrival patterns and behaviour, such as oviposition, of sarcosaphagous insects through the production of metabolically derived volatile organic compounds^44^,^45^,^46^. The interactions between insects and microbial communities are still poorly understood in carrion decomposition systems.

Decomposition rates depend on the extent of internal and external environmental interactions. Because of the predictable succession of corpse-colonizing sarcosaphagous arthropods and their specific life stages, insects are regularly used as important indicators of PMI in terrestrial environments, especially in corpses displaying advanced decay^47^. However, insects may arrive at a carcass several hours to several days after death, which might lead to imprecise estimates of PMI. In addition, insect activity can be inhibited by rain and low temperatures, and can be absent from some decomposing remains^48^. Therefore, microbial communities might offer an additional biometric for PMI estimation, as the succession of microbial community structures is a continuous process rather than a series of separate processes^47^. Several studies also confirmed that bacterial communities can be used as a microbial clock for assisting in estimates of PMI^25^,^26^. The current study supports these previous findings and provides additional data. Our study also demonstrated that the abundance of dominant taxa was not significantly altered by the presence or absence of sarcosaphagous insects, meaning that microbial community structure could be applied as an indicator of PMI in various situations. Additionally, knowing how the bacterial community structure changes in response to insect arrival and colonization patterns of carrion might modify the accuracy of PMI estimation by sarcosaphagous arthropods. However, there are a variety of challenges for researchers studying microbial communities during decomposition. High-throughput sequencing is too expensive for routine analysis and other forensic techniques have required considerable testing and refinement long after they were initially conceived^49^. Therefore, whilst forensic microbiology offers enormous potential to be developed into a standard and widely accepted forensic tool for PMI estimation, there is a need for a great deal of basic research before this can be realized.

## Methods

### Study sites and sample grouping

To study the bacterial community during decomposition, we used adult female Sprague Dawley rat carcasses (n = 18) as models of human decomposition. The experiments were conducted in Changsha City, Hunan Province, central south China (28.12°N, 112.58°E) in June 2014. The rats, each weighting 200–220 g, were randomly divided into two groups and killed by blunt force causing spinal cord transection. Each carcass was individually placed into a sterile cardboard box (0.5 × 0.3 × 0.25 m) lined with a thick layer of sterile sawdust to absorb the subsequent putrefactive liquid without surrounding contamination. In the sarcosaphagous insect access group (group B), the boxes were left open to allow insect access. In the sarcosaphagous insect exclusion group (group A), each box was placed in a fine mesh grenadine bag (60 mesh/inch) to allow normal air circulation but exclude insects. At approximately 1300 GMT on 1 June, 2014, the boxes containing carcasses were randomly placed at least 5 m apart on the ground in an area dominated by herbaceous plants and cherry trees. We observed physical changes in the carcasses each day, and recorded the number, species and distribution of insects in the sarcosaphagous insect access group. The eggs, larvae and adult insects were collected three times per day (0700, 1300 and 1900 GMT), and some of the immature insects were raised to adulthood for morphological taxonomic identification, and for sequencing of a fragment of the cytochrome oxidase subunit I gene region, as described previously^16^. WTH0T1-3-0.5 intelligent temperature and humidity loggers (Wangyunshan Information Technology Co., Fuzhou, China) were placed within 0.6 m of each carcass, approximately 0.3 m above the ground. Ambient air temperature and humidity were monitored hourly during the course of the experiments. The study was approved by the Medical Ethics Committee of Xiangya Hospital Central South University (approval code: 201303147), and the methods were carried out in accordance with the approved guidelines.

### Bacterial community sample collection

Bacterial communities were sampled prior to death (less than 1 min before killing), immediately after death (less than 10 min post-mortem), and then at 4 h, 12 h, 1 day, 2 days, 3 days, 4 days, 6 days and 8 days post-mortem (sample names are provided in Table 1). Two body sites, the buccal cavity and rectum, were swabbed lightly for 60 s using sterile cotton applicators premoistened with sterile ultrapure water. Once the corpses reached the stage of advanced decay, the buccal cavity and rectum were no longer discernible in animals from group B, and sampling ended after day 3 post-mortem. For group A, buccal cavity sampling was no longer possible after 6 days post-mortem, meaning that only the rectum was sampled at the 8-day time point. After sampling, the tip of the applicator was cut off and placed in a 1.5-mL microcentrifuge tube containing 1 mL sterile ultrapure water. All samples were immediately frozen at −20 °C until further processing (within 24 h).

### DNA extraction, PCR amplification and next-generation sequencing

Genomic DNA was extracted using a MoBio PowerSoil DNA Isolation Kit (Mo Bio Laboratories, Carlsbad, CA, USA), following the manufacturer’s specifications. DNA concentration and purity were checked on 1% agarose gels, and DNA was diluted to a 1 ng/μL working stock. DNA samples from the nine rat carcasses in each group at each time point were mixed in equal concentrations, and the mixed DNA specimens were sent to Novogene Biological Information Technology Co. (Beijing, China) for analysis by MiSeq sequencing.

PCR reactions were conducted using the 515F/806R primer set^50^, which amplifies the V4 region of the 16S rRNA gene (forward primer: 5′-GTGCCAGCMGCCGCGGTAA-3′, reverse primer: 5′-GGACTACHVGGGTWTCTAAT-3′). The reverse primer contained a 6-bp error-correcting barcode unique to each sample. All PCR reactions were carried out in 30-μL reaction volumes containing 15 μL of Phusion High-Fidelity PCR Master Mix (New England Biolabs, Ipswich, MA, USA), 0.2 μM forward and reverse primers, and 10 ng template DNA. Thermal cycling conditions were: 98 °C for 1 min, followed by 30 cycles of 98 °C for 10 s, 50 °C for 30 s, and 72 °C for 30 s, with a final extension at 72 °C for 5 min. Resulting amplicons were confirmed on 2% agarose gels containing ethidium bromide.

All amplicons were in the size range of 400–450 bp, and were purified using a GeneJET Gel Extraction Kit (Thermo Fisher Scientific, Carlsbad, CA, USA). Following quantitation, equal concentrations of the purified amplicons were combined into a single tube. Sequencing libraries were generated using a NEB Next Ultra DNA Library Prep Kit for Illumina (New England Biolabs, Ipswich, MA, USA) following manufacturer’s recommendations, and index codes were added. The library quality was assessed on a Qubit 2.0 Fluorometer (Thermo Fisher Scientific, Carlsbad, CA, USA) and Agilent Bioanalyzer 2100 system. Sequencing was conducted on an Illumina MiSeq platform, which generated 300-bp paired-end reads.

### Data analysis

Paired-end reads from the original DNA fragments were merged using FLASH version 1.2.7^51^. After merging, sequences were processed and analysed using QIIME version 1.7.0^52^. Sequences containing ambiguous bases (N) or low-quality bases were filtered out using QIIME filter^53^, and chimeras were removed using UCHIME^54^. Remaining sequences were assigned to each sample according to the unique barcodes, and the sequences were rarefied prior to statistical analyses at the level of 9200.

Clustering was performed using the UPARSE pipeline (version 7.0.1001)^27^, and similar sequences were assigned to OTUs using the threshold of 97% identity. A representative sequence was picked from each OTU by selecting the longest sequence that had the largest number of hits to other sequences in the OTU. The RDP classifier (version 2.2)^55^ was used to annotate taxonomic information for each representative sequence. The representative sequences were aligned using the Greengenes database^56^, with a minimum identity of 80%. The phylogenetic relationships of representative sequences were determined using PyNAST software (Version 1.2)^57^, and the GreenGenes database “Core Set” data^58^ was used for rapid multiple sequence alignment. “Rare taxa” was used to describe the taxa with a relative abundance of less than 1% of the total abundance of all taxa in all samples, while the unclassified reads were grouped into “Others”.

Non-linear regression between bacterial taxon richness (OTU numbers, Chao1, Observed Species and Shannon index values) and ADH was performed using PASW Statistical Software version 18 (SPSS Ltd, Quarry Bay, Hong Kong). ADHs were a summation of temperature (°C) above the lower development threshold multiplied by hours^15^. Because ADH was used to explore how bacterial communities (not insects) changed in the current study, a lower development threshold of 0 °C was employed in this calculation^26^.

Alpha diversity was estimated using three phylogenetic diversity metrics: Chao1, Observed Species and Shannon index. Rarefaction curves were generated based on Observed Species. Beta diversity between bacterial communities was evaluated using both weighted and unweighted unifrac distances^59^. Hierarchical clustering of samples was completed using UPGMA. PCoA on the unifrac distances of the unweighted and weighted distance matrices was performed to visualise differences in bacterial community composition and structure, respectively. Analysis of similarities description^60^ was performed based on the Bray–Curtis dissimilarity distance matrices to test the differences in community composition among groups of samples.

## Additional Information

**How to cite this article**: Guo, J. *et al*. Potential use of bacterial community succession for estimating post-mortem interval as revealed by high-throughput sequencing. *Sci. Rep.* **6**, 24197; doi: 10.1038/srep24197 (2016).

## Supplementary Material

Supplementary Information

## Figures and Tables

**Table 1:** Operational taxonomic unit (OTU)-based diversity indexes in rat buccal cavity and rectum samples during decomposition.

Sample name	Sample group	Sample region	Sample time	Raw sequences	Clean sequences	OTUs	Chao 1	Observed Species	Shannon index
AM01	group A	buccal cavity	alive	44201	43698	179	237.83	123	2.53
AM02	group A	buccal cavity	0 h	25848	23616	830	884.56	599	5.47
AM03	group A	buccal cavity	4 h	58277	55573	753	517.39	308	4.08
AM04	group A	buccal cavity	12 h	40267	39830	250	192.63	133	2.50
AM05	group A	buccal cavity	1 d	51774	51340	161	131.09	77	1.20
AM06	group A	buccal cavity	2 d	52858	52179	209	140.05	103	3.03
AM07	group A	buccal cavity	3 d	39238	38500	313	256.44	167	3.48
AM08	group A	buccal cavity	4 d	32886	31310	725	632.90	512	5.58
AM09	group A	buccal cavity	6 d	52732	51566	258	199.56	114	2.56
AA01	group A	rectum	alive	43810	42253	417	464.97	323	3.81
AA02	group A	rectum	0 h	38292	36672	403	399.37	346	4.60
AA03	group A	rectum	4 h	36413	35332	315	278.11	247	3.21
AA04	group A	rectum	12 h	31063	29971	323	271.57	248	3.78
AA05	group A	rectum	1 d	44670	43274	339	338.67	250	4.02
AA06	group A	rectum	2 d	61996	61368	181	131.04	104	1.44
AA07	group A	rectum	3 d	38890	38473	159	149.96	121	1.75
AA08	group A	rectum	4 d	15137	14982	99	148.58	99	1.52
AA09	group A	rectum	6 d	31587	31347	101	119.11	74	1.62
AA10	group A	rectum	8 d	59499	58837	171	155.33	127	1.70
BM01	group B	buccal cavity	alive	49263	48589	199	149.32	118	3.05
BM02	group B	buccal cavity	0 h	54850	51060	994	827.82	522	4.98
BM03	group B	buccal cavity	4 h	19412	19034	195	172.69	137	3.35
BM04	group B	buccal cavity	12 h	9611	9201	393	375.38	363	4.89
BM05	group B	buccal cavity	1 d	90467	89315	295	190.05	125	1.89
BM06	group B	buccal cavity	2 d	43100	42579	196	181.77	109	2.18
BM07	group B	buccal cavity	3 d	19340	18706	262	263.88	171	2.99
BA01	group B	rectum	alive	75480	71302	437	382.94	314	4.96
BA02	group B	rectum	0 h	20778	19587	307	287.66	250	2.79
BA03	group B	rectum	4 h	48171	45954	394	348.72	290	3.48
BA04	group B	rectum	12 h	48059	45838	350	326.03	259	2.97
BA05	group B	rectum	1 d	26588	25769	324	373.84	270	3.06
BA06	group B	rectum	2 d	47855	47275	211	171.58	130	1.31
BA07	group B	rectum	3 d	61768	60987	234	159.37	129	2.17

**Table 2:** Shared phyla among the buccal cavity and rectum communities from samples collected immediately before and after death (less than 10 min).

Phylum	Shared OTUs	Shared reads				Shared OTUs	Shared reads			
		AM01	AM02	BM01	BM02		AA01	AA02	BA01	BA02
Actinobacteria	7	894	770	1002	988	1	8	4	2	1
Bacteroidetes	3	3	35	5	25	57	10900	9166	7793	9631
Deferribacteres	0	0	0	0	0	1	4	37	903	4
Firmicutes	24	3705	2106	3844	2272	78	2615	4531	4369	4210
Proteobacteria	21	4437	3193	4181	3530	19	578	361	1138	730
Tenericutes	1	1	18	4	33	0	0	0	0	0
Verrucomicrobia	0	0	0	0	0	1	67	122	243	112
Total shared reads	56	9040	6122	9036	6848	157	14172	14221	14448	14688
Total reads		9175	8810	9177	8792		14940	14985	14943	14950
Shared reads/Total reads (%)		98.53	69.49	98.46	77.89		94.86	94.90	96.69	98.25

OTU: operational taxonomic unit. Sample names are as defined in Table 1.
