# Supplementary material for: Potential use of bacterial community succession for estimating post-mortem interval as revealed by high-throughput sequencing
Source: Sci Rep. 2016 Apr 7;6:24197. doi: 10.1038/srep24197 (PMC4823735; doi:10.1038/srep24197)
Supplement: Supplementary Information [file srep24197-s1.pdf]

Potential use of bacterial community succession for estimating post-mortem interval as revealed by high-throughput sequencing

Juanjuan Guo[1], Xiaoliang Fu[1], Huidan Liao[1], Zhenyu Hu[2], Lingling Long[1], Weitao Yan[1], Yanjun Ding[1], Lagabaiyila Zha[1], Yadong Guo[1], Jie Yan[1], Yunfeng Chang[1], Jifeng Cai[1]*

Supplementary Table S1

Succession of sarcosaphagous insects attracted to the various stages of exposed rat carcasses in Changsha.

Order	Family	Species	Fresh			Bloated			Active			Advanced			Dry		
			A	L	E	A	L	E	A	L	E	A	L	P	A	L	P
Diptera	Calliphoridae	*Lucilia cuprina*	√			√	√	√	√	√	√	√	√			√	√
		Lucilia sericata	√			√	√	√	√	√	√	√	√		√	√	√
		Chrysomya pinguis	√			√		√	√	√		√	√			√	√
		Hemipyrellia ligurriens							√			√					
		Achoerandrus rufifacies	√			√		√	√		√	√	√			√	√
	Sarcophagidae	*Boettcherisca peregrine*				√			√								
		Parasarcophaga crassipalpis							√			√					
		Phallosphaera gravelyi				√			√			√					
	Muscidae	*Musca domestica*	√			√			√			√			√		
		Hydrotaea chalcogaster							√			√			√		
	Phoridae	*Unident*				√			√		√		√				√
Coleoptera	Staphylinidae	*Creophilus maxillosus*										√			√		
	Silphidae	*Calosilpha brunneicollis*							√			√			√		
	Nitidulidae	*Omosita discoidea*										√			√		
	Dermestidae	*D. maculatus Degeer*													√		

A, adult; L, larva; E; egg; P, pupa.

Supplementary Table S2

Numbers of adult dipterans collected at different stages of decomposition from exposed rat carcasses

Family	Species	Fresh	Bloated	Active	Advanced	Dry
Calliphoridae	*Lucilia cuprina*	6	11	24	27	0
	Lucilia sericata	4	17	20	19	7
	Chrysomya pinguis	7	15	13	21	0
	Hemipyrellia ligurriens	0	0	8	15	0
	Achoerandrus rufifacies	13	23	27	31	0
Sarcophagidae	*Boettcherisca peregrine*	0	2	2	1	0
	Parasarcophaga crassipalpis	0	0	2	4	0
	Phallosphaera gravelyi	0	4	5	5	0
Muscidae	*Musca domestica*	5	9	7	11	2
	Hydrotaea chalcogaster	0	0	5	5	4
Phoridae	*Unident*	0	10	26	0	0

Supplementary Table S3

Genera with significantly different abundance in the buccal cavity between the sarcosaphagous insect exclusion group (group A) and the sarcosaphagous insect access group (group B).

**Taxa**	**mean abundance (group A)**	**mean abundance (group B)**	***p* value**
Escherichia	0.0015649	0.0054790	0.0091
Wautersiella	0.0000120	0.0009286	0.0163
Collinsella	0.0000120	0.0001703	0.0463
Deinococcus	0.0000963	0	0.0114
Methylocaldum	0.0000843	0	0.0209
Dok59	0.0000963	0	0.0114
Photobacterium	0	0.0000774	0.0160
Spirochaeta	0.0000722	0	0.0387
SargSea-WGS	0	0.0000774	0.0160
Halanaerobium	0.0001204	0	0.0401
Facklamia	0	0.0000774	0.0160
Acholeplasma	0.0000722	0	0.0387

Supplementary Table S4

Genera with significantly different abundance in the rectum between the sarcosaphagous insect exclusion group (group A) and the sarcosaphagous insect access group (group B).

Taxa	mean abundance (group A)	mean abundance (group B)	*p* value
Stenotrophomonas	0.0000542	0.0005237	0.0005
Anaerovibrio	0.0204442	0.0032864	0.0402
Sutterella	0.0018310	0.0088299	0.0432
Thiobacillus	0	0.0000903	0.0074
Wautersiella	0	0.0001625	0.0013
Collinsella	0.0012026	0.0004695	0.0338
Bifidobacterium	0.0000542	0.0001806	0.0469
Sulfurimonas	0	0.0000722	0.0198
Clostridium	0.0001733	0	0.0129
Adlercreutzia	0.0000975	0	0.0169
Caulobacter	0.0000867	0	0.0289
Lachnospira	0	0.0000722	0.0198

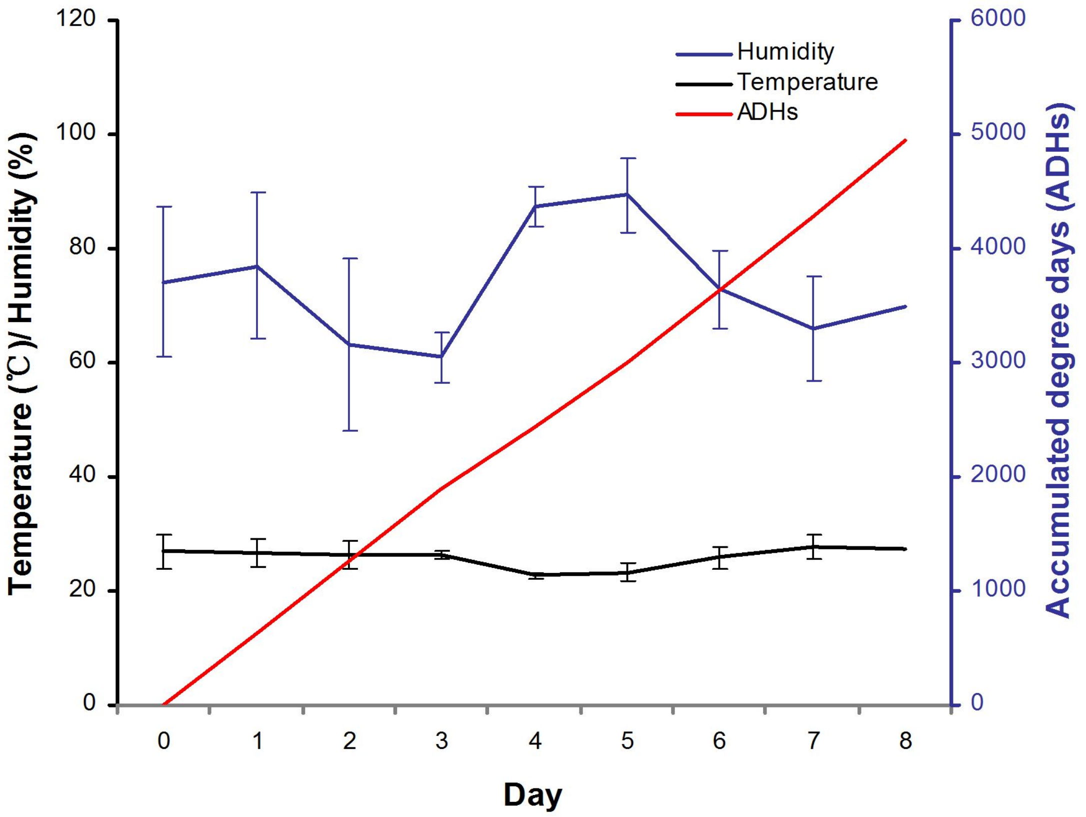

Supplementary Figure S1

Average daily temperature (°C), humidity (%) and accumulated degree hours (ADHs) during the experiment

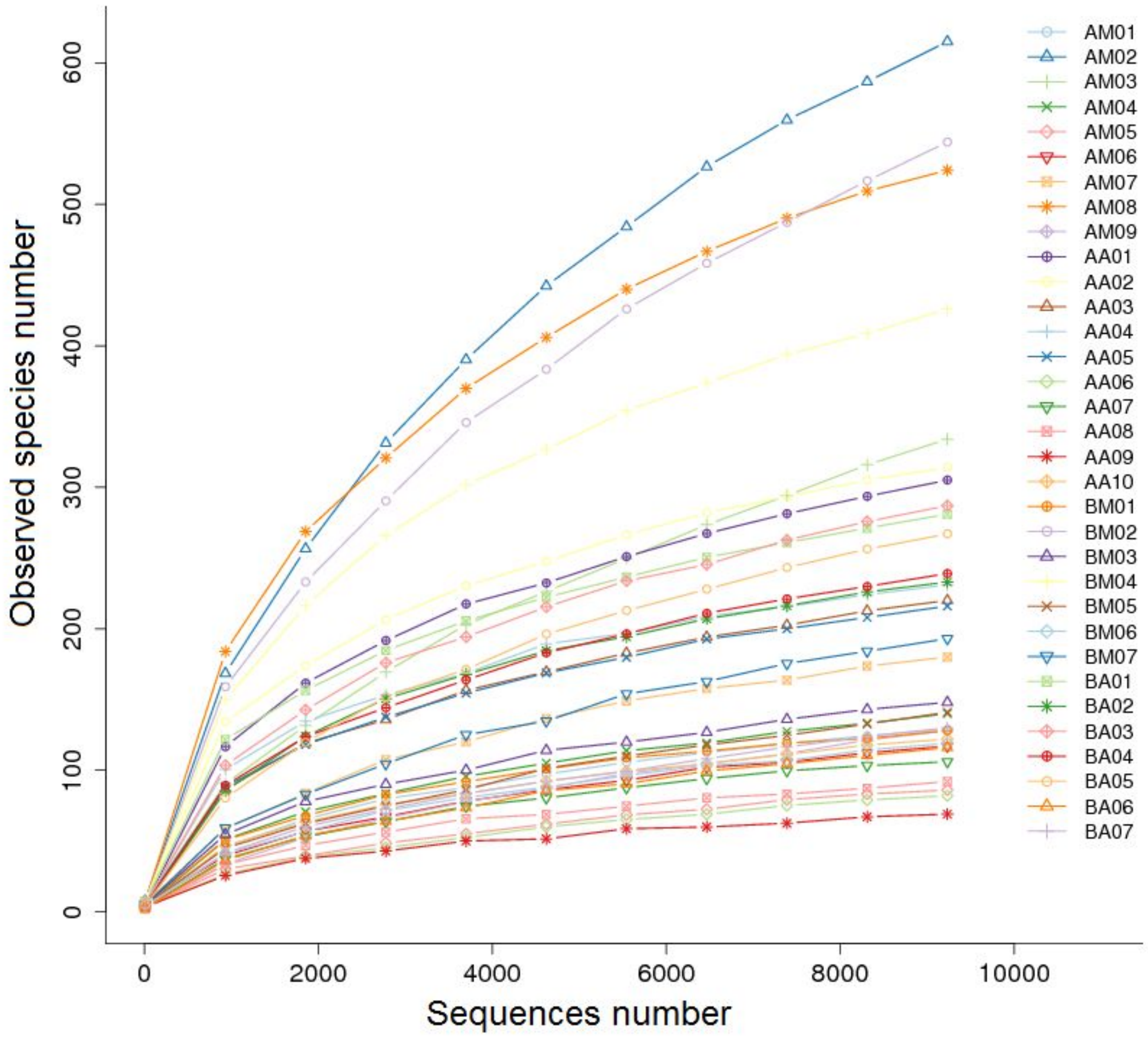

Supplementary Figure S2

Rarefaction curves of observed species number clustered at 97% sequence identity across all samples. Sample names refer to samples as described in Table 1.

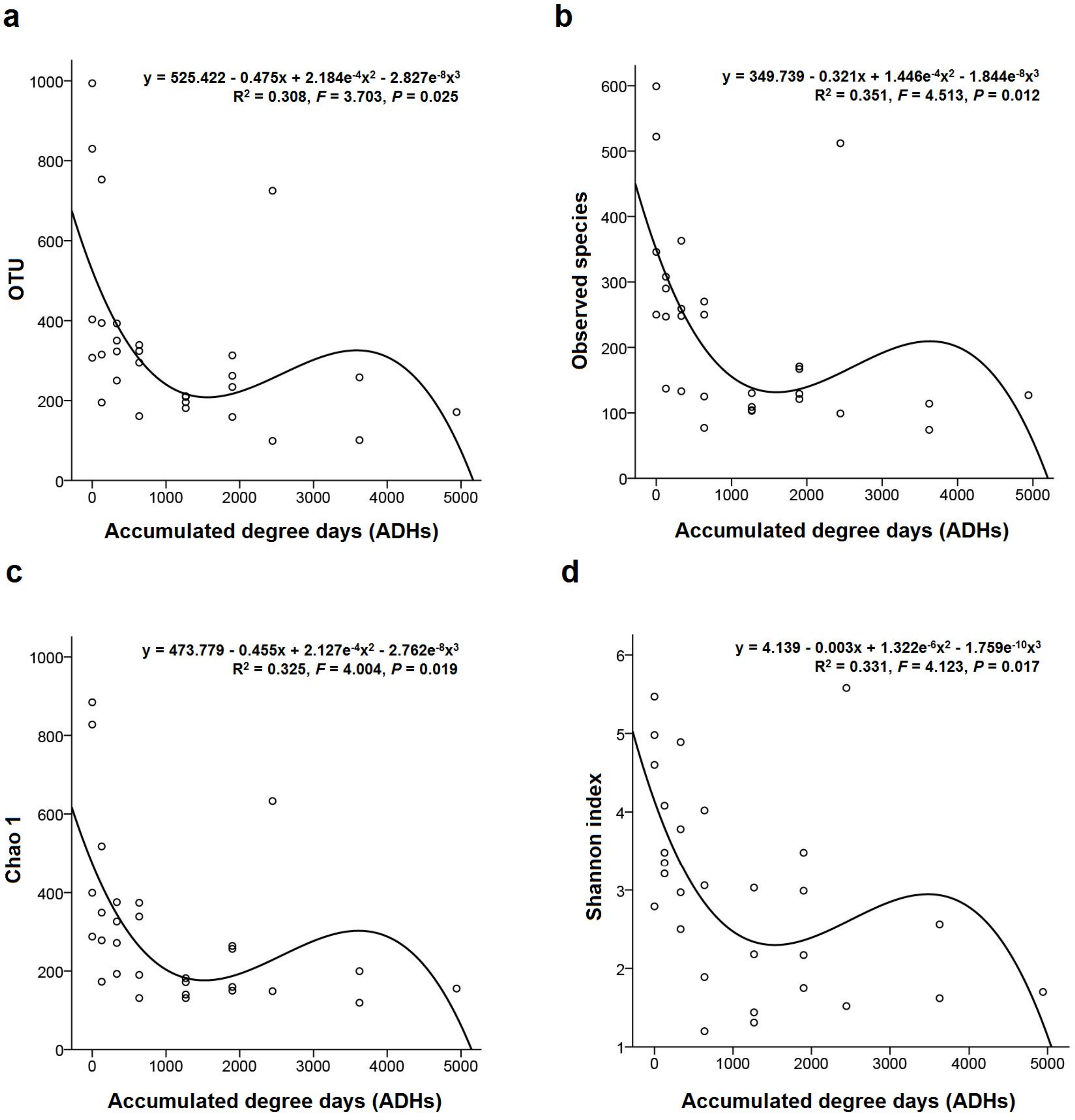

Supplementary Figure S3

Non-linear regression curves of operational taxonomic unit (OTU) richness (a), observed species index (b), Chao1 index (c) and Shannon index (d) during decomposition. Each open circle represents the bacterial community of a single sampling body site (i.e., buccal cavity or rectum) from rat carcasses.

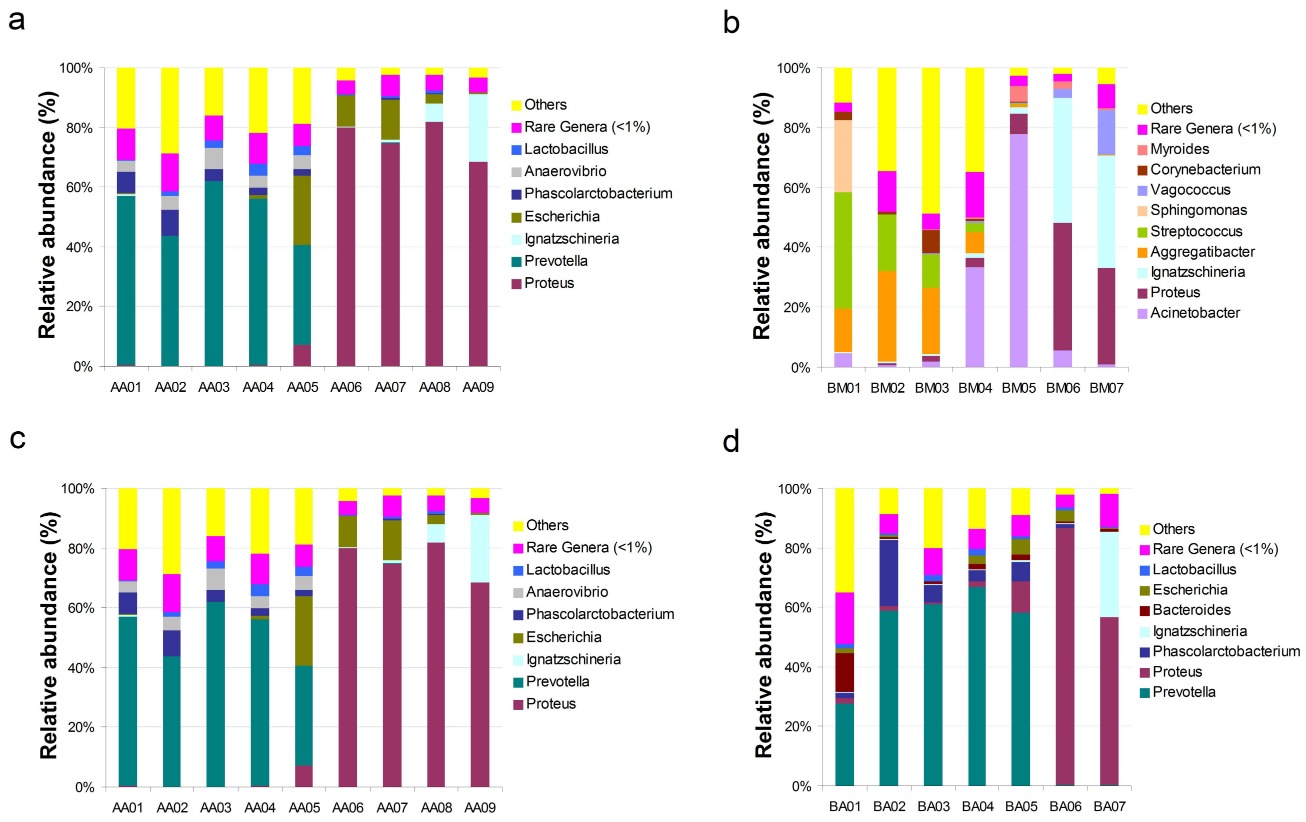

Supplementary Figure S4

Bacterial community structure variation during decomposition at the genus level. Relative abundance of bacterial genera during decomposition in the buccal cavity in group A (a), buccal cavity in group B (b), rectum in group A (c) and rectum in group B (d). Sample names refer to samples as described in Table 1.

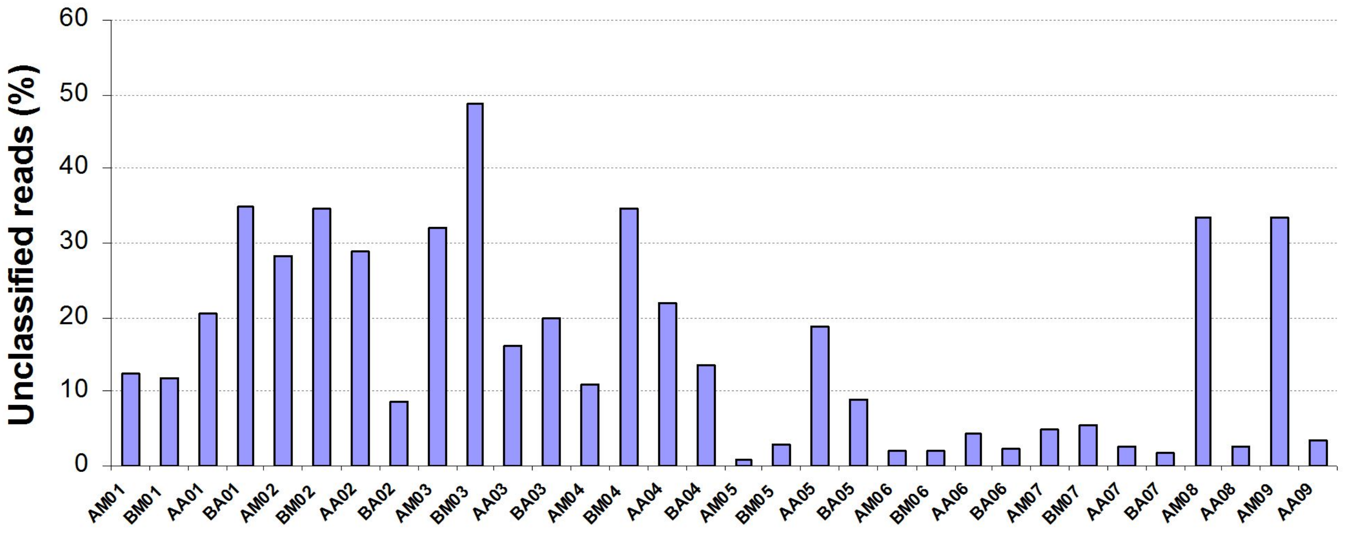

Supplementary Figure S5

Proportion of the unclassified reads in different samples. Sample names refer to samples as described in Table 1.
